# Fixed BMI eligibility criteria for GLP-1 receptor agonist trials and estimated trial-eligible proportions in Asian and non-Asian populations: A cross-sectional analysis

**DOI:** 10.1371/journal.pone.0351415

**Published:** 2026-06-25

**Authors:** Ki Young Huh, Ildae Song

**Affiliations:** 1 Department of Clinical Pharmacology and Therapeutics, Seoul National University College of Medicine and Hospital, Seoul, Republic of Korea; 2 Healthcare AI Research Institute, Seoul National University Hospital, Seoul, Republic of Korea; 3 Department of Pharmaceutical Science and Technology, Kyungsung University, Busan, Republic of Korea; The Chinese University of Hong Kong, HONG KONG

## Abstract

Glucagon-like peptide-1 receptor agonists (GLP-1 RAs) are globally developed for metabolic diseases, yet clinical trials have underrepresented Asian populations who develop metabolic complications at lower body mass index (BMI) values than Non-Asian populations. This cross-sectional study characterized eligibility criteria in 352 GLP-1 RA trials registered on ClinicalTrials.gov (2017–2020) and estimated the proportion of populations meeting these criteria using nationally representative health survey data from 23,251 adults (5,984 Non-Asian US, 353 Asian US, and 16,914 Korean) from National Health and Nutrition Examination Survey (NHANES, US) and Korean NHANES (2021–2023). Mean BMI was substantially higher in Non-Asian US adults (29.8 kg/m^2^) compared with Asian US (24.9 kg/m^2^) and Korean (24.2 kg/m^2^) populations. BMI criteria, specified in 233 trials (66.2%), demonstrated the largest eligibility disparities. For trials requiring BMI ≥ 30 kg/m^2^, eligibility was 41.5% for Non-Asian US versus 13.5% for Asian US and 7.2% for Korean adults. In contrast, HbA1c, eGFR, and liver function criteria showed minimal between-population differences (all > 93% meeting typical thresholds). These findings suggest that fixed BMI eligibility criteria are associated with substantially lower estimated trial-eligible proportions among Asian populations, supporting further evaluation of ethnicity-sensitive BMI thresholds in future GLP-1 RA trial design.

## Introduction

Glucagon-like peptide-1 receptor agonists (GLP-1 RAs) have emerged as important therapeutic options for metabolic diseases [1]. Originally approved for glycemic control in type 2 diabetes, these agents have shown clinically meaningful efficacy in weight management [2]. The cardiovascular benefits of GLP-1 RAs have expanded beyond populations with diabetes, as evidenced by the SELECT trial showing a 20% reduction in major adverse cardiovascular events among patients with obesity without diabetes [3]. These advances have established GLP-1 RAs as one of the most promising therapeutic classes in metabolic medicine [4].

Despite the expanding therapeutic landscape of GLP-1 RAs, the underrepresentation of diverse populations in clinical trials remains a concern [5]. Asian populations exhibit distinct anthropometric and metabolic profiles: at equivalent BMI values, Asian individuals have 3–5% higher body fat percentage, greater visceral adiposity, and higher risks for metabolic syndrome than Caucasians [6–9]. 21–44% of Asian individuals with “normal BMI” exhibit two or more metabolic abnormalities [10].

However, clinical trial eligibility criteria for GLP-1 RAs have largely employed uniform thresholds across ethnic groups, without accounting for these differences [11]. The International Council for Harmonisation (ICH) E5 and E17 guidelines advocate for evaluation of ethnic factors that may affect drug response [12,13]. However, systematic implementation of these principles in trial design remains inconsistent. Recent analyzes have documented significant underrepresentation of racial and ethnic minorities in GLP-1 RA clinical trials, with White participants comprising 66.6–93.2% of major cardiovascular outcome trials and Asian participants only 1.2–21.6% [5,14]. The use of fixed BMI thresholds that do not account for ethnic differences may contribute to this underrepresentation, as fewer Asian individuals meet Western-derived cutoffs for obesity despite being at comparable or higher metabolic risk [15].

In this cross-sectional study, we examined the eligibility criteria specified in GLP-1 RA clinical trials and estimated the proportion of US and Korean populations meeting these criteria. Using data from ClinicalTrials.gov and nationally representative health surveys from the United States (National Health and Nutrition Examination Survey, NHANES) and Korea (Korean National Health and Nutrition Examination Survey, KNHANES), we aimed to characterize the distribution of quantitative eligibility criteria in GLP-1 RA trials registered between 2017 and 2020. In addition, we estimated the proportion of non-Asian US, Asian US, and Korean populations meeting these criteria to evaluate the inclusiveness of current eligibility criteria across diverse populations.

## Materials and methods

### Study design and data sources

This cross-sectional study analyzed publicly available data from three sources: ClinicalTrials.gov for GLP-1 receptor agonist trial eligibility criteria, the US National Health and Nutrition Examination Survey (NHANES) 2021−2023, and the Korea National Health and Nutrition Examination Survey (KNHANES) 2021−2023. The study period was selected to reflect the recent expansion of GLP-1 RA indications and to align with overlapping survey cycles with comparable laboratory assays between NHANES and KNHANES. This study followed the Strengthening the Reporting of Observational Studies in Epidemiology (STROBE) reporting guideline for cross-sectional studies [16].

Clinical trial data were obtained from the Aggregate Analysis of ClinicalTrials.gov (AACT) database [17]. We included GLP-1 receptor agonist trials registered between January 1, 2017, and December 31, 2020. Trial identification was performed using keyword matching for names of GLP-1 receptor agonists (exenatide, liraglutide, dulaglutide, semaglutide, tirzepatide, lixisenatide, albiglutide) and drug class keywords (GLP-1, glucagon-like peptide-1, incretin). Data extracted included study phase, eligibility criteria text, intervention details, and study start dates

The NHANES is a continuous, nationally representative survey of the noninstitutionalized civilian US population conducted by the National Center for Health Statistics (NCHS), Centers for Disease Control and Prevention. The survey uses a complex, multistage probability sampling design, with oversampling of certain subgroups to increase precision of estimates for these populations. NHANES 2021–2023 (August 2021 to August 2023) data were used for this analysis [18]. The survey consists of household interviews and standardized physical examinations conducted at mobile examination centers, including laboratory measurements of glycemic, lipid, and kidney function parameters.

The KNHANES is an annual, nationally representative cross-sectional survey of the noninstitutionalized civilian Korean population conducted by the Korea Disease Control and Prevention Agency (KDCA) [19,20]. The survey employs a complex, stratified, multistage probability cluster sampling design based on population and housing census data. Combined data from KNHANES IX comprising surveys in 2021, 2022, and 2023 were used to increase statistical power. The survey includes health interviews, physical examinations, and laboratory tests.

### Study population

For the analysis of U.S. populations, adults aged 18 years or older with valid Mobile Examination Center (MEC) examination weights were included from the NHANES dataset. Participants were classified into two groups based on self-reported race/ethnicity: Asian (Non-Hispanic Asian) and non-Asian (Mexican American, Other Hispanic, Non-Hispanic White, Non-Hispanic Black, and Other Race/Multiracial).

For the analysis of the Korean population, adults aged 19 years or older (the legal age of majority in Korea) with valid sampling weights were included from the KNHANES dataset. Given the ethnic homogeneity of the Korean population, all participants were considered comparable to Asian Americans for the purposes of this analysis.

Clinical trials registered on *ClinicalTrials.gov* between January 1, 2017, and December 31, 2020, were included if they involved at least one GLP-1 receptor agonist intervention and had available eligibility criteria text. Trials were classified by phase: early phase (Phase 1, Phase 1/2, and Phase 2) and late phase (Phase 2/3, Phase 3, and Phase 4). The geographical distribution of trial conduct was characterized using the AACT countries table.

Clinical trial eligibility criteria were extracted from trials registered between 2017 and 2020 to capture the period of expanded GLP-1 RA indications. Population data from NHANES and KNHANES 2021–2023 were used because the NHANES 2019–2020 cycle was incomplete due to the COVID-19 pandemic, and the 2021–2023 cycle represents the most recent complete survey with comparable laboratory assays across both countries. Population-level anthropometric and metabolic characteristics are relatively stable over short time intervals, supporting the application of contemporary population data to evaluate trial eligibility criteria from this period.

### Ethics statement

This study used publicly available, deidentified data from three sources. The NHANES 2021–2023 protocol was approved by the National Center for Health Statistics Ethics Review Board (Protocol #2021-05 for NHANES 2021–2023). The KNHANES IX (2021–2023) protocol was approved by the Korea Disease Control and Prevention Agency Institutional Review Board (2018-01-03-5C-A, 2018-01-03-4C-A, 2022-11-16-R-A for KNHANES 2021–2023); written informed consent was obtained from all participants. Clinical trial data were obtained from the publicly accessible Aggregate Analysis of ClinicalTrials.gov database. This secondary analysis of deidentified public data was determined to be exempt from review by the Seoul National University Hospital Institutional Review Board, and the requirement for informed consent was waived because the analysis used deidentified, publicly available data. All publicly available datasets were accessed for research purposes between January 02 and January 31, 2026. The authors did not have access to information that could identify individual participants during or after data collection, as all datasets were deidentified prior to public release.

### Eligibility criteria and primary endpoint extraction

Quantitative eligibility criteria were extracted from free-text fields using a two-stage approach: keyword-based regular expression matching to identify relevant text segments, and numerical value extraction with context-aware parsing to determine cutoff values and comparison operators. The extracted results were manually curated by clinical experts for accuracy. Criteria extracted included: demographics (age, sex), anthropometric measures (BMI, body weight), glycemic parameters (HbA1c, fasting plasma glucose), kidney function (eGFR), hepatic function (AST, ALT), and blood pressure. For criteria with range specifications (e.g., BMI 25–40 kg/m^2^), both minimum and maximum values were extracted. When multiple patterns matched, the most specific pattern was retained. Details of the extraction algorithm are provided in Supplementary S1 Table. AI-assisted tools (Claude, Anthropic) were used for automated parsing and extraction of eligibility criteria from clinical trial registry free-text fields, with subsequent expert curation and validation. The authors reviewed, verified, and take full responsibility for all content.

To address population-relevant exclusion criteria, we screened the eligibility-criteria text of all 352 trials for five categories using keyword-based screening followed by independent duplicate review and clinical expert adjudication. Each candidate hit was classified as a true exclusion criterion, a target indication (inclusion), or not mentioned. The five categories were non-alcoholic or metabolic-associated fatty liver disease (non-alcoholic fatty liver disease, non-alcoholic steatohepatitis, metabolic-associated fatty liver disease, or metabolic-associated steatohepatitis); severe hepatic impairment (cirrhosis, hepatic failure, hepatitis B or C-positive serology, alanine aminotransferase or aspartate aminotransferase ≥2.5 times the upper limit of normal); monogenic forms of diabetes (maturity-onset diabetes of the young, mutations in hepatocyte nuclear factor 1-alpha, hepatocyte nuclear factor 4-alpha, or glucokinase genes, secondary diabetes); prior bariatric or weight-loss surgery; and explicit race or ethnicity restrictions.

Primary endpoints for each trial were extracted from the AACT database and classified into mutually exclusive principal categories: glycated hemoglobin change, other glycemic measures, body weight in kilograms, body weight as percent change, other weight measures, body mass index change, waist circumference, liver or non-alcoholic steatohepatitis outcomes, renal outcomes, cardiovascular outcomes, pharmacokinetic and pharmacodynamic measures, safety and adverse events, quality-of-life or patient-reported outcomes, and other. Classification was performed using an initial automated pass followed by clinical expert adjudication.

### Variables

Variables were defined using standard criteria (Supplementary S2 Table). BMI was calculated as weight in kilograms divided by height in meters squared. Elevated waist circumference was defined using population-specific criteria. Glycated hemoglobin, fasting glucose, lipids, eGFR, liver enzymes, and blood pressure were measured using standardized methods.

### Outcomes and covariates

For each eligibility criterion, trials were categorized based on extracted numeric cutoffs and their direction (minimum, maximum, or range). Trials without explicit quantitative thresholds were classified by qualitative descriptors (e.g., ‘T2D diagnosis required’, ‘Liver disease excluded’, ‘Not specified’). The primary outcome was the proportion of each population (non-Asian US, Asian US, and Korean) meeting specific eligibility criteria, calculated as weighted prevalence using complex survey methods. The distribution of eligibility criteria across GLP-1 RA trials was calculated as the number and percentage of trials specifying each quantitative criterion. Subgroup analyzes were performed by study phase (early phase [Phase 1, Phase 1/2, Phase 2] vs late phase [Phase 2/3, Phase 3, Phase 4]) and by study drug (semaglutide, tirzepatide, liraglutide, dulaglutide, exenatide, lixisenatide, and others). Trials using generic GLP-1 RA class terms without specifying a particular drug were classified as ‘GLP-1 RA (generic)’. Trials involving investigational or less common GLP-1 RAs (e.g., albiglutide, orforglipron, retatrutide) were classified as ‘Others’.

### Statistical analysis

All analyzes accounted for the complex survey designs of NHANES and KNHANES using appropriate sampling weights to produce nationally representative estimates. For NHANES, MEC examination weights were applied with masked variance pseudo-primary sampling units and pseudo-strata. For KNHANES, sampling weights were applied with primary sampling units and strata.

Continuous variables were summarized as weighted means with standard deviations and 95% CIs. Categorical variables were summarized as weighted proportions with 95% CIs. Differences across populations were assessed using survey-weighted chi-square tests for categorical variables and survey-weighted ANOVA for continuous variables. P-values < .05 were considered statistically significant.

Weighted prevalence of meeting individual eligibility criteria was estimated for each population. Eligibility rates were stratified by study drug and study phase, with differences assessed using survey-weighted ANOVA. Tukey’s method with Bonferroni correction was applied for post-hoc pairwise comparisons. Co-occurrence patterns between eligibility criteria were visualized using heatmaps. Pairwise correlations between eligibility variables were calculated.

Sequential eligibility attrition was analyzed by applying criteria in a predefined order and calculating the cumulative proportion of the population remaining eligible at each step. This analysis was performed separately for two representative trial types: type 2 diabetes trials (age 18–75 years, BMI ≥ 25 kg/m^2^, HbA1c 7.0–10.0%, eGFR ≥ 30 mL/min/1.73m^2^) and obesity trials (age 18–65 years, BMI ≥ 27 kg/m^2^ and BMI ≥ 30 kg/m^2^ (to illustrate progressive attrition), HbA1c < 6.5%, eGFR ≥ 60 mL/min/1.73m^2^). For Korean participants, the minimum age criterion was set at 19 years to reflect the legal age of majority in Korea.

All statistical analyzes were performed using R version 4.5.2 (R Foundation for Statistical Computing) with the *survey* package for complex survey analyzes.

## Results

### Baseline characteristics

A total of 5,984 Non-Asian US adults, 353 Asian US adults, and 16,914 Korean adults were included in the analysis. Sex distribution was comparable across populations, with approximately 50% female in each group (*P* = .17). However, age distribution differed significantly (*P* < .001): Asian US participants were younger (mean 43.4 years, SD 16.9) compared with Non-Asian US (48.0 years, SD 18.1) and Korean (49.1 years, SD 16.9) participants.

**Fig 1:**
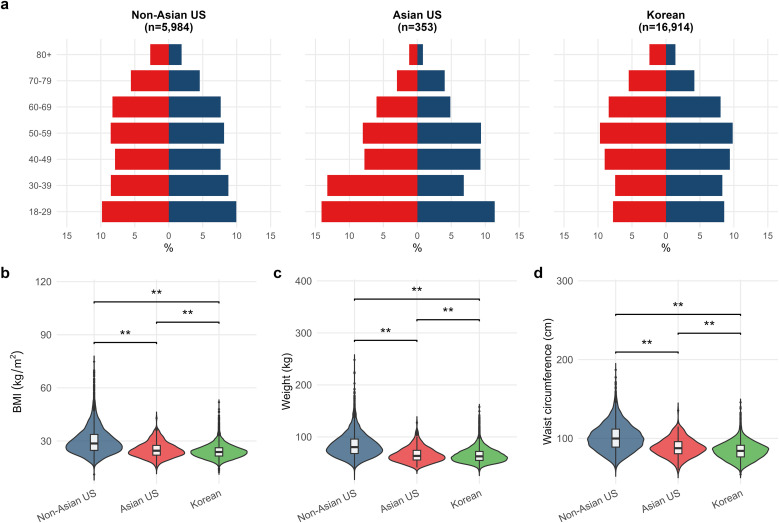
Participant characteristics. Demographic and anthropometric characteristics of study populations from NHANES (2021-2023, United States) and KNHANES (2021-2023, Korea). **(A)** Age and sex distribution shown as population pyramids for Non-Asian US (n = 5,984), Asian US (n = 353), and Korean (n = 16,914) adults. Blue bars represent males; red bars represent females. The x-axis shows the percentage of each population within age groups. Asian US participants were younger (mean 43.4 years) compared with Non-Asian US (48.0 years) and Korean (49.1 years) participants. **(B)** Body mass index (BMI) distribution. Non-Asian US adults showed substantially higher BMI (mean 29.8 kg/m^2^) compared with Asian US (24.9 kg/m^2^) and Korean (24.2 kg/m^2^) populations. **(C)** Body weight distribution. Mean weight was 84.6 kg in Non-Asian US, 67.0 kg in Asian US, and 66.3 kg in Korean adults. **(D)** Waist circumference distribution. Non-Asian US adults had larger waist circumference (mean 100.7 cm) compared with Asian US (88.5 cm) and Korean (84.1 cm) populations. Violin plots show the distribution shape; white dots indicate medians; thick bars indicate interquartile ranges. **P < .001 for between-group comparisons.

Anthropometric measures showed marked differences between populations (all *P* < .001). Mean BMI was substantially higher in Non-Asian US adults (29.8 kg/m^2^, SD 7.4) compared with Asian US (24.9 kg/m^2^, SD 4.4) and Korean (24.2 kg/m^2^, SD 3.8) participants. Correspondingly, the proportion with BMI ≥ 30 kg/m^2^ was 41.5% in Non-Asian US versus 13.5% in Asian US and 7.2% in Korean populations (Table 1, Fig 1).

**Table 1:** Characteristics of study participants.

Characteristic	Non-Asian US(n = 5984)	Asian US(n = 353)	Korean(n = 16914)	*P* Value
**Age, y**	48.0 (18.1)	43.4 (16.9)	49.1 (16.9)	<.001
**Female, %**	51.4	53.4	50.3	.17
**BMI, kg/m** ^**2**^	29.8 (7.4)	24.9 (4.4)	24.2 (3.8)	<.001
Underweight (<18.5), %	1.7	4.3	4.4	<.001
Normal (18.5–24.9), %	25.2	50.7	58.4	
Overweight (25.0–29.9), %	31.6	31.6	30.0	
Obese I (30.0–34.9), %	21.1	11.0	5.9	
Obese II (35.0–39.9), %	10.4	2.3	1.0	
Obese III (>=40.0), %	9.9	0.1	0.2	
**Body weight, kg**	84.6 (22.6)	67.0 (14.9)	66.3 (13.9)	<.001
**Height, cm**	168.3 (10.1)	163.5 (9.5)	165.1 (9.4)	<.001
**Waist circumference, cm**	100.7 (17.2)	88.5 (11.6)	84.1 (11.1)	<.001
Elevated ^a^	59.4	26.1	35.8	<.001
**HbA1c, %**	5.7 (1.1)	5.7 (1.0)	5.6 (0.8)	.15
<5.7%	66.2	66.6	65.6	.002
5.7-6.4%	23.6	25.4	25.9	
>=6.5%	10.2	8.0	8.5	
**Fasting glucose, mg/dL**	108.6 (35.5)	109.6 (52.6)	100.8 (22.9)	<.001
Normal (<100), %	48.3	52.2	63.7	<.001
Prediabetes (100–125), %	40.2	36.6	28.5	
Diabetes (>=126), %	11.5	11.1	7.8	
**Total cholesterol, mg/dL**	187.2 (42.5)	192.8 (40.0)	189.4 (39.4)	.07
>=240 mg/dL, %	9.9	11.4	9.6	.74
**HDL cholesterol, mg/dL**	53.6 (14.4)	54.3 (13.8)	55.4 (14.8)	<.001
Low HDL ^b^	26.4	21.4	22.3	.01
**LDL cholesterol, mg/dL**	109.1 (36.0)	111.5 (36.7)	115.2 (36.1)	<.001
>=160 mg/dL, %	4.1	5.2	7.4	<.001
**Triglycerides, mg/dL**	120.8 (101.4)	117.1 (59.5)	129.2 (102.5)	.004
>=200 mg/dL, %	5.4	5.4	13.0	<.001
**eGFR** ^c^**, mL/min/1.73m²**	95.6 (21.3)	104.7 (18.1)	101.5 (16.7)	<.001
G1 (>=90), %	64.5	84.2	79.4	<.001
G2 (60–89), %	29.1	12.6	18.6	
G3a (45–59), %	4.6	2.6	1.4	
G3b (30–44), %	1.3	0.5	0.4	
G4 (15–29), %	0.3	0.0	0.2	
G5 (<15), %	0.1	0.1	0.0	
**ALT, U/L**	22.5 (17.5)	24.1 (17.2)	23.5 (21.0)	.01
>40 U/L, %	7.2	10.2	9.9	<.001
**AST, U/L**	22.6 (14.8)	22.4 (10.8)	23.5 (13.6)	.002
>40 U/L, %	3.8	3.5	5.1	.001
**Systolic BP, mmHg**	120.9 (17.0)	118.9 (16.8)	118.6 (15.6)	<.001
**Diastolic BP, mmHg**	74.4 (11.0)	74.7 (10.1)	74.0 (9.9)	.26
Hypertension (>=140/90), %	15.8	11.7	11.8	<.001
**Self-reported hypertension, %**	30.8	25.0	23.0	<.001
**Self-reported dyslipidemia, %**	35.0	29.6	21.2	<.001
**Laboratory-defined dyslipidemia** ^d^	37.0	32.9	36.4	.55

Data are presented as weighted mean (SD) for continuous variables and weighted percentage for categorical variables.

^a^Elevated waist circumference defined as ≥102 cm for men and ≥88 cm for women in the US populations (NCEP ATP III criteria) and ≥90 cm for men and ≥85 cm for women in the Korean population (Korean Society for the Study of Obesity criteria).

^b^Low HDL cholesterol defined as <40 mg/dL for men and <50 mg/dL for women.

^c^eGFR calculated using the CKD-EPI 2021 equation without race coefficient.

^d^Laboratory-defined dyslipidemia defined as total cholesterol ≥240 mg/dL, LDL cholesterol ≥160 mg/dL, low HDL cholesterol, or triglycerides ≥200 mg/dL.

Abbreviations: ALT, alanine aminotransferase; AST, aspartate aminotransferase; BMI, body mass index; BP, blood pressure; CKD-EPI, Chronic Kidney Disease Epidemiology Collaboration; eGFR, estimated glomerular filtration rate; HbA1c, glycated hemoglobin; HDL, high-density lipoprotein; LDL, low-density lipoprotein; NCEP ATP III, National Cholesterol Education Program Adult Treatment Panel III.

Despite the large sample sizes, glycemic parameters showed modest clinical differences across populations. Mean HbA1c was similar across groups (5.7%, 5.7%, and 5.6% for Non-Asian US, Asian US, and Korean, respectively; *P* = .15). The prevalence of diabetes (HbA1c ≥ 6.5%) was 10.2% in Non-Asian US, 8.0% in Asian US, and 8.5% in Korean populations. However, fasting plasma glucose differed (*P* < .001), with Korean adults showing lower mean levels (100.8 mg/dL, SD 22.9) compared with Non-Asian US (108.6 mg/dL, SD 35.5) and Asian US (109.6 mg/dL, SD 52.6). Baseline characteristics are summarized in Table 1, with population distributions shown in Fig 1 and Supplementary S1 Fig.

### Trial eligibility criteria and endpoints

Among the 352 GLP-1 RA trials analyzed, eligibility criteria varied substantially in specification and thresholds (Table 2). Age criteria were the most commonly specified, appearing in 346 trials (98.3%). The upper age limit demonstrated differential effects across populations: trials with age ≤ 65 years included 79.4% of Non-Asian US adults versus 86.9% of Asian US adults (*P* = .01), reflecting the younger age distribution of Asian participants.

**Table 2:** Eligibility criteria specified in GLP-1 receptor agonist trials.

Variable	Trials, No. (%)	Non-Asian US	Asian US	Korean	*P* Value
**Age, y**	346 (98.3)				
No upper limit	117 (33.2)	100.0	100.0	100.0	
18-65	35 (9.9)	79.4	86.9	81.0	.01
18-75	37 (10.5)	92.4	96.2	92.6	.02
**BMI**	233 (66.2)				
≥23	21 (6.0)	85.2	64.7	59.3	<.001
≥25	32 (9.1)	73.1	45.0	37.1	<.001
≥27	42 (11.9)	59.7	32.1	20.1	<.001
≥30	22 (6.2)	41.5	13.5	7.2	<.001
≥35	4 (1.1)	20.4	2.5	1.2	<.001
No lower limit	14 (4.0)	98.8	96.9	97.2	<.001
Maximum only specified	14 (4.0)	–	–	–	
**Body weight, kg**	136 (38.6)				
≥50	30 (8.5)	97.8	89.5	90.4	<.001
Stable weight required	97 (27.6)	–	–	–	
Other specifications	9 (2.6)	–	–	–	
**HbA1c**	187 (53.1)				
≥5.7 (prediabetes)	3 (0.9)	33.8	33.4	34.4	.91
≥6.5 (diabetes)	21 (6.0)	10.2	8.0	8.5	.002
≥7.0 (uncontrolled)	56 (15.9)	7.0	6.0	4.8	<.001
7.0-10.0 (range)	16 (4.5)	5.5	4.0	4.3	.007
Maximum only specified	73 (20.7)	–	–	–	
T2D diagnosis required	183 (52.0)	–	–	–	
Non-diabetic or prediabetes	9 (2.6)	–	–	–	
**Fasting plasma glucose, mg/dL**	51 (14.5)				
≥100 (prediabetes)	12 (3.4)	51.7	47.8	36.3	<.001
≥126 (diabetes)	11 (3.1)	11.5	11.1	7.8	<.001
Other specifications	28 (8.0)	–	–	–	
**Triglycerides, mg/dL**	34 (9.7)				
<400	5 (1.4)	98.4	100.0	98.1	<.001
Other specifications	29 (8.2)	–	–	–	
**eGFR, mL/min/1.73m** ^**2**^	139 (39.5)				
≥30	54 (15.3)	99.6	99.9	99.8	.006
≥45	11 (3.1)	98.2	99.4	99.4	<.001
≥60	51 (14.5)	93.6	96.8	98.0	<.001
Renal impairment excluded	34 (9.7)	–	–	–	
Normal function required	23 (6.5)	–	–	–	
**ALT, U/L**	104 (29.5)				
≤3 × ULN	50 (14.2)	99.3	99.1	99.0	.44
≤2.5 × ULN	29 (8.2)	99.0	98.2	98.4	.22
≤2 × ULN	15 (4.3)	98.1	96.8	97.2	.13
Liver disease excluded	138 (39.2)	–	–	–	
**Systolic BP, mm Hg**	64 (18.2)				
<160	33 (9.4)	97.3	96.2	98.1	<.001
<180	16 (4.5)	99.3	99.4	99.7	.21
Uncontrolled HTN excluded	30 (8.5)	–	–	–	
Controlled BP required	6 (1.7)	–	–	–	
**Diastolic BP, mm Hg**	62 (17.6)				
<100	27 (7.7)	98.3	97.7	98.6	.10
<90	19 (5.4)	91.4	93.4	93.5	.03
Uncontrolled HTN excluded	30 (8.5)	–	–	–	
Controlled BP required	16 (4.5)	–	–	–	

Population eligibility calculated using NHANES 2021–2023 (Non-Asian US, Asian US) and KNHANES 2021–2023 (Korea) data with survey weights are calculated. Percentages represent the proportion of each population meeting the specified criterion. Categories without numeric cutoffs (marked with –) were not included in eligibility calculations or in the total count for each variable.

Abbreviations: ALT, alanine aminotransferase; BMI, body mass index; BP, blood pressure; eGFR, estimated glomerular filtration rate; FPG, fasting plasma glucose; HbA1c, glycated hemoglobin; HTN, hypertension; T2D, type 2 diabetes; ULN, upper limit of normal.

BMI criteria were specified in 233 trials (66.2%), with substantial variation in minimum thresholds. The proportion of eligible participants declined steeply at higher BMI cutoffs among Asian populations. For example, trials requiring BMI ≥ 25 kg/m^2^ would include 73.1% of Non-Asian US adults but only 45.0% of Asian US and 37.1% of Korean adults (*P* < .001). At the BMI ≥ 30 kg/m^2^ threshold, eligibility dropped to 41.5%, 13.5%, and 7.2% for Non-Asian US, Asian US, and Korean populations, respectively (*P* < .001). Body weight criteria, specified in 136 trials (38.6%), showed similar patterns but with smaller absolute differences between populations.

HbA1c criteria were specified in 187 trials (53.1%). Prediabetes thresholds (HbA1c ≥ 5.7% or ≥6.0%) did not significantly differ in eligibility rates across populations. However, diabetes-specific criteria showed greater disparity: trials requiring HbA1c ≥ 7.0% would include 7.0% of Non-Asian US adults versus 6.0% of Asian US and 4.8% of Korean adults (*P* < .001). Fasting plasma glucose was specified in only 51 trials (14.5%), and triglyceride criteria in 34 trials (9.7%).

In contrast to BMI criteria, kidney function (eGFR), hepatic function (ALT), and blood pressure criteria showed minimal between-population differences in eligibility rates. For typical exclusion thresholds, more than 93% of all populations met eGFR, ALT, and blood pressure criteria (Table 2). Eligibility criteria co-occurrence patterns are shown in Supplementary Figs S2 and S3.

The 352 trials were conducted across more than 50 countries; country information was available for 344 (97.7%). The most frequent locations were the United States (141 trials, 40.1%), Germany (53, 15.1%), China (49, 13.9%), Canada (38, 10.8%), the United Kingdom (37, 10.5%), Japan (28, 8.0%), and the Republic of Korea (14, 4.0%); 95 trials (27.0%) included at least one site in an East-, South-, or Southeast-Asian country (S4 Table).

Across the 352 trials, severe hepatic impairment was an exclusion criterion in 177 trials (50.3%), prior bariatric or weight-loss surgery in 111 (31.5%), and monogenic forms of diabetes (MODY, HNF1A/HNF4/GCK, or ‘secondary diabetes’) in 33 (9.4%). Six trials (1.7%) restricted enrollment to specific racial or ethnic groups in a way that functions as an exclusion of others. NAFLD/NASH/MAFLD/MASH was never used as an exclusion criterion; instead, it appeared as the target indication in 21 trials (6.0%), in which biopsy- or imaging-confirmed steatohepatitis was a required inclusion criterion (S5 Table).

HbA1c change was the principal primary endpoint in 76 trials (21.6%), other glycemic measures (fasting plasma glucose, insulin or C-peptide, time in range, diabetes remission) in 37 (10.5%), and body-weight-related measures in 37 (10.5%), comprising percent body-weight change in 26 trials (7.4%), absolute weight change in kilograms in 6 (1.7%), and other weight measures in 5 (1.4%). Other principal endpoints included pharmacokinetic or pharmacodynamic measures (42, 11.9%), safety and adverse events (47, 13.4%), cardiovascular outcomes (28, 8.0%), liver or NASH outcomes (11, 3.1%), quality-of-life or patient-reported outcomes (6, 1.7%), renal outcomes (6, 1.7%), BMI change (1, 0.3%), and a heterogeneous ‘other’ category (61, 17.3%) covering disease-specific endpoints (S6 Table).

Among the 352 trials, individual components of the T2D composite criteria depicted in Fig 3 (age 18–75, BMI ≥ 25 kg/m^2^, HbA1c 7.0–10.0%, eGFR ≥ 30 mL/min/1.73m^2^) were specified in 196 trials (55.7%) for age maximum ≤75, 108 (30.7%) for BMI ≥ 25, 82 (23.3%) for HbA1c minimum ≥7.0% (with 29 trials, 8.2%, specifying the full HbA1c 7.0–10.0% range), and 130 (36.9%) for eGFR ≥ 30; the full four-component set was specified together in 6 trials (1.7%). For the obesity composite (age 18–65, BMI ≥ 30, HbA1c < 6.5%), components were specified individually in 109 trials (31.0%) for age maximum ≤65, 27 (7.7%) for BMI ≥ 30, and 7 (2.0%) for HbA1c < 6.5%; all three components together appeared in 1 trial (0.3%). Among trials with a numeric minimum BMI, the WHO Asia–Pacific cutoff of ≥23 kg/m^2^ was specified by 21 trials (6.0%), ≥ 25 by 32 (9.1%), ≥ 27 by 42 (11.9%), and ≥30 by 22 (6.2%) (Table 2; full breakdown in S7 Table).

When the obesity composite (age 18–65, BMI ≥ 30, HbA1c < 6.5%, eGFR ≥ 60) was applied separately by sex, the population-level disparity (Non-Asian US > Asian US > Korean) was preserved in both sexes. The Non-Asian US estimate was 27.2% in men versus 28.6% in women; for Asian US adults, 11.4% versus 8.2%; for Korean adults, 6.8% versus 4.2%. For T2D-trial eligibility, the corresponding estimates were 4.5% (M) versus 3.8% (F) in Non-Asian US, 1.8% versus 1.3% in Asian US, and 2.6% versus 1.6% in Korean adults. Within-population sex differences were small and inconsistent in direction (S8 Table).

### Eligibility rates by drug, trial phase, and population

Eligibility rates varied by GLP-1 RA drug and trial phase (Supplementary S3 Table, S4 Fig). Early phase trials (Phase 1, 1/2, 2; n = 143) generally showed higher and more uniform eligibility rates compared with late phase trials (Phase 2/3, 3, 4; n = 141). Mean eligibility in early phase trials was 44.8% (SD 27.7) for Non-Asian US, 41.2% (SD 29.4) for Asian US, and 37.0% (SD 30.1) for Korean populations. In contrast, late phase trials showed lower overall eligibility: 31.9% (SD 32.2), 26.3% (SD 31.7), and 23.1% (SD 32.1), respectively.

**Fig 2:**
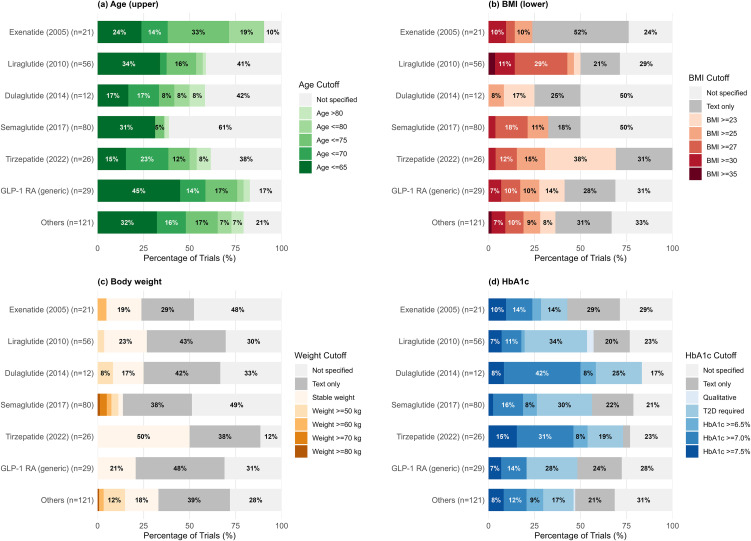
Eligibility criteria distribution by GLP-1 receptor agonist drug. Distribution of key eligibility criteria (age, body mass index [BMI], glycated hemoglobin [HbA1c], and estimated glomerular filtration rate [eGFR]) across 352 GLP-1 receptor agonist clinical trials, stratified by drug. Each panel shows the minimum and/or maximum thresholds specified in trial protocols. Horizontal lines within violin plots indicate median values. Drugs are ordered by FDA approval year (earliest to most recent): exenatide (2005), liraglutide (2010), dulaglutide (2014), lixisenatide (2016), semaglutide (2017), and tirzepatide (2022). BMI minimum thresholds show the greatest variability, with most trials specifying cutoffs between 25 and 35 kg/m^2^. Age criteria typically range from 18 to 75 years, while HbA1c criteria (when specified) most commonly require values between 7.0% and 10.0%.

Drug-specific patterns emerged across trial phases (Fig 2). Among early phase trials, exenatide studies showed the highest mean eligibility rates (60.6%, 62.5%, and 64.4% for Non-Asian US, Asian US, and Korean, respectively), followed by liraglutide (54.0%, 47.5%, 40.3%). Tirzepatide early phase trials showed lower eligibility (39.5%, 35.4%, 31.3%) with high variability (SD 28.9–29.6%), reflecting the diversity of early phase study designs.

In late phase trials, dulaglutide studies showed the highest eligibility rates (42.7%, 43.3%, 42.9% across populations), while semaglutide trials showed moderate eligibility (33.5%, 26.3%, 23.5%). Tirzepatide late phase trials demonstrated very low overall eligibility: 12.0% for Non-Asian US, 7.3% for Asian US, and 5.1% for Korean populations.

**Fig 3:**
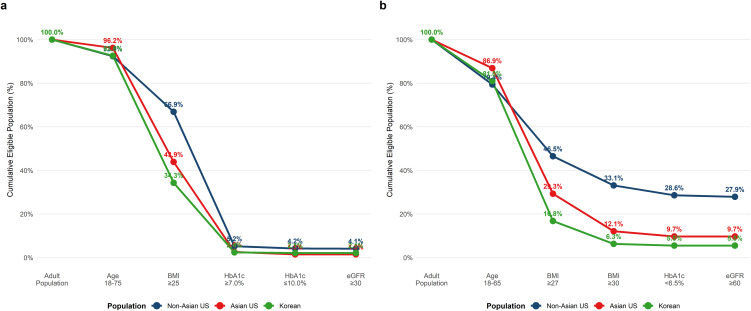
Sequential eligibility attrition by composite trial criteria. Waterfall analysis showing sequential application of eligibility criteria for (A) type 2 diabetes trials and (B) obesity trials across three populations: Non-Asian US, Asian US, and Korean adults. For type 2 diabetes trials, criteria were applied in sequence: age 18-75 years (19-75 for Korea), BMI ≥ 25 kg/m^2^, HbA1c 7.0-10.0%, and eGFR ≥ 30 mL/min/1.73m^2^. For obesity trials: age 18-65 years (19-65 for Korea), BMI ≥ 27 kg/m^2^, BMI ≥ 30 kg/m^2^, HbA1c < 6.5%, and eGFR ≥ 60 mL/min/1.73m^2^. In type 2 diabetes trials, the HbA1c requirement (7.0-10.0%) resulted in uniformly low eligibility across all populations. In obesity trials, the BMI ≥ 30 kg/m^2^ criterion represented the largest source of eligibility attrition in Asian populations, producing substantial between-population disparities in final eligibility.

Sequential application of composite eligibility criteria revealed differential attrition patterns between trial types (Fig 3). For type 2 diabetes trials applying typical criteria (age 18–75 years, BMI ≥ 25 kg/m^2^, HbA1c 7.0–10.0%, eGFR ≥ 30 mL/min/1.73m^2^), final eligibility was uniformly low across populations: 4.1% for Non-Asian US, 1.5% for Asian US, and 2.1% for Korean adults, with the HbA1c requirement representing the primary exclusion criterion. In contrast, obesity trials applying fixed criteria (age 18–65 years, BMI ≥ 30 kg/m^2^, HbA1c < 6.5%) demonstrated marked between-population disparities: final eligibility was 27.9% for Non-Asian US versus 9.7% for Asian US and 5.5% for Korean adults. The BMI ≥ 30 kg/m^2^ criterion accounted for the largest attrition in Asian populations, reducing eligibility from 79.4–86.9% to 12.1–16.8% at this single step.

## Discussion

In this cross-sectional analysis of GLP-1 receptor agonist clinical trials and nationally representative survey data from the United States and Korea, we found substantial differences in trial eligibility rates across ethnic populations. BMI emerged as the most critical determinant of eligibility disparities: using the standard BMI ≥ 30 kg/m^2^ threshold for obesity trials, only 13.5% of Asian US adults and 7.2% of Korean adults would be eligible, compared with 41.5% of Non-Asian US adults. These findings suggest that current GLP-1 RA trial designs may contribute to lower estimated trial-eligible proportions among Asian populations, which could in turn limit the generalizability of trial results to these groups.

BMI-based eligibility criteria reflect regulatory frameworks established primarily from Caucasian populations [21]. However, Asian individuals develop type 2 diabetes, metabolic syndrome, and cardiovascular disease at substantially lower BMI values, supporting lower thresholds of ≥23 kg/m^2^ for obesity screening in these populations [9,22,23]. Our findings quantify how fixed BMI thresholds may exclude Asian individuals who could meet metabolic-risk criteria for GLP-1 RA treatment. As shown in Results, individual components of the typical T2D and obesity composite criteria were widely employed, although the full composite sets were specified together in only 6 (1.7%) and 1 (0.3%) trials, respectively. This indicates that fixed BMI cutoffs operate primarily through their pervasive individual use rather than as a single rigid bundle.

The heterogeneity within Asian populations also warrants consideration. Our study revealed demographic and metabolic differences between Asian Americans and Koreans, including higher triglyceride levels in Koreans despite the association between US residence and cardiometabolic abnormalities [24,25]. These findings suggest that multinational trials should account for both demographic and cultural factors when designing eligibility criteria.

Trial-design considerations also constrain eligibility-criteria choice. Continuous anthropometric endpoints, such as change in body weight (kg or %), BMI, and waist circumference, together with HbA1c-based glycemic endpoints, accounted for the principal primary endpoint of most efficacy trials in our cohort (S6 Table). These endpoints typically require recruiting participants whose baseline values fall within a relatively narrow range to achieve adequate statistical power. Together with regulatory frameworks anchored in non-Asian reference populations, these constraints may contribute to the predominance of fixed BMI thresholds observed in our analysis and to the resulting eligibility disparities across populations.

While most population-relevant exclusions are not population-specific in their wording, the higher prevalence of hepatic steatosis reported in some Asian subgroups, combined with the very high prevalence of severe-hepatic-impairment exclusion criteria (50.3% of trials), may exert differential impact in practice (S5 Table).

We note that NHANES (United States) and KNHANES (Korea) were used in this study as nationally representative reference frames against which the trial eligibility criteria were tested, rather than as the actual enrollment cohorts of the 352 trials, which are distributed across more than 50 countries (S4 Table). The multi-country footprint of the included trials supports our rationale that ethnicity-sensitive eligibility criteria, rather than a single fixed BMI threshold, may be needed to support equitable representation across populations whose anthropometric and metabolic profiles differ from the reference frames historically used to design these trials.

Our findings support consideration of ethnicity-specific BMI thresholds, which may increase estimated eligibility among Asian populations while better aligning enrollment with metabolic risk profiles. For obesity trials, using BMI ≥ 25 kg/m^2^ for Asian populations would increase Korean eligibility from 7.2% to 37.1% [26]. This approach aligns with ICH E5 and E17 guidelines advocating evaluation of ethnic factors affecting drug response [13]. The Korean Society for the Study of Obesity guidelines, which define obesity at BMI ≥ 25 kg/m^2^, provide a precedent for region-specific thresholds [27]. Recent trials such as STEP 11 have adopted Asian-specific BMI thresholds, demonstrating feasibility [28]. Additionally, supplementary measures such as body composition should be considered [21,29].

This study has several limitations. First, the temporal gap between trial registration (2017–2020) and population data (2021–2023) may affect eligibility estimates, although population-level anthropometric characteristics are relatively stable over short intervals. Second, the small sample size of Asian US participants (n = 353) may limit precision of estimates for this subgroup. Third, race/ethnicity in NHANES was self-reported, which may introduce misclassification. Fourth, although NHANES is a US-based sample, the trials we analyzed were conducted across many countries (most commonly the United States, Germany, and China); NHANES and KNHANES were therefore used as nationally representative reference frames rather than as enrollment cohorts (S4 Table). Fifth, sex-stratified analyses (S8 Table) confirmed that population-level disparities in estimated eligibility were preserved in both sexes, with small and inconsistent within-population sex differences.

This analysis adds to the limited literature quantifying the differential impact of GLP-1 RA trial eligibility criteria across Asian and non-Asian populations using multiple nationally representative datasets. Future investigations should consider body composition measures such as waist-to-height ratio and incorporate ethnicity-specific criteria from the outset of clinical development.

## Conclusions

In this cross-sectional analysis of GLP-1 receptor agonist trials and nationally representative surveys from the US and Korea, fixed BMI eligibility criteria were associated with substantially lower estimated trial-eligible proportions among Asian compared with non-Asian populations. These findings support further evaluation of ethnicity-sensitive BMI or adiposity-based eligibility criteria in future GLP-1 RA trial design.

## Supporting information

S1 Table Hybrid methodology for structured eligibility criteria extraction. (DOCX)

S2 Table Measurements and laboratory methods. (DOCX)

S3 Table Population eligibility rates by trial phase and drug. (DOCX)

S4 Table Country distribution of the 352 GLP-1 RA trials. (DOCX)

S5 Table Population-relevant exclusion criteria after manual review of the eligibility-criteria text of all 352 GLP-1 RA trials. (DOCX)

S6 Table Primary-endpoint classification of the 352 GLP-1 RA trials. (DOCX)

S7 Table Distribution of minimum BMI eligibility thresholds across the 352 GLP-1 RA trials. (DOCX)

S8 Table Sex-stratified estimated trial-eligible proportions in NHANES and KNHANES adults, by population. (DOCX)

S1 Fig Distribution of laboratory parameters across populations. Violin plots showing the distribution of key laboratory parameters across three populations: Non-Asian US (blue), Asian US (red), and Korean (green) adults. Parameters include glycated hemoglobin (HbA1c), fasting plasma glucose, body mass index (BMI), estimated glomerular filtration rate (eGFR), triglycerides, LDL cholesterol, and alanine aminotransferase (ALT). Horizontal dashed lines indicate common eligibility thresholds used in GLP-1 receptor agonist clinical trials. White dots within violins represent median values; thick bars represent interquartile ranges. Survey weights were applied to generate population-representative distributions. Notable differences include substantially higher BMI distributions in Non-Asian US compared with Asian populations, and higher triglyceride levels in Korean adults.
(PNG)

S2 Fig Co-occurrence of eligibility criteria in GLP-1 RA clinical trials. Heatmap showing the percentage of 352 trials in which pairs of eligibility criteria were specified together. Diagonal values (gray) represent the percentage of trials specifying each individual criterion: age (98.3%), BMI (66.2%), HbA1c (53.1%), eGFR (39.5%), liver function (29.5%), blood pressure (18.2%), fasting plasma glucose (11.9%), body weight (11.1%), and lipids (9.7%). Off-diagonal values represent co-occurrence rates. Criteria are ordered by frequency (descending). P values were calculated using chi-squared tests. Abbreviations: ALT, alanine aminotransferase; BMI, body mass index; eGFR, estimated glomerular filtration rate; FPG, fasting plasma glucose; GLP-1 RA, glucagon-like peptide-1 receptor agonist; HbA1c, glycated hemoglobin.
(PNG)

S3 Fig Correlation matrix of eligibility criteria across populations. Three-panel heatmap showing tetrachoric correlations between binary eligibility criteria across Non-Asian US, Asian US, and Korean populations. Data are from NHANES 2021−2023 (Non-Asian US and Asian US) and KNHANES 2021−2023 (Korean). Strong positive correlations were observed between HbA1c ≥ 6.5% and HbA1c ≥ 7.0% (r = 0.73–0.80) and between BMI ≥ 25 and BMI ≥ 30 (r = 0.35–0.50), indicating that related criteria track together across populations. Moderate negative correlations were observed between age criteria (18−65 years) and hypertension (r = −0.24 to −0.33), reflecting the age-dependent nature of hypertension prevalence. eGFR criteria (≥30 and ≥60 mL/min/1.73m^2^) showed consistent positive correlations (r = 0.26–0.27) across all three populations.
(PNG)

S4 Fig Population eligibility rates by trial phase and GLP-1 RA drug. Box plots showing mean population eligibility rates stratified by trial phase (Early Phase: Phase 1/2, n = 143; Late Phase: Phase 3/4, n = 141) and specific GLP-1 receptor agonist drug. Eligibility rates were calculated using NHANES 2021–2023 (Non-Asian US, Asian US) and KNHANES 2021–2023 (Korean) data with survey weights. Early phase trials showed higher mean eligibility (44.8% Non-Asian US) compared to late phase trials (31.9% Non-Asian US), reflecting less restrictive criteria in earlier development stages. Among late phase trials, Lixisenatide (n = 5) showed the lowest eligibility rates (5.8% Non-Asian US) due to strict HbA1c requirements (7.5–10%), while Dulaglutide (n = 7) showed the highest variability (SD = 46.2%). Tirzepatide late phase trials (n = 10) showed low eligibility (12.0% Non-Asian US) as the SURPASS program focused exclusively on type 2 diabetes with strict glycemic thresholds. Trials with phase not specified or not applicable were excluded from this analysis.
(DOCX)

## References

[1] Moiz A, Filion KB, Tsoukas MA, Yu OHY, Peters TM, Eisenberg MJ. The expanding role of GLP-1 receptor agonists: a narrative review of current evidence and future directions. EClinicalMedicine. 2025;86:103363. doi: 10.1016/j.eclinm.2025.103363 40727007 PMC12303005

[2] Mozaffarian D. GLP-1 agonists for obesity — a new recipe for success?. JAMA. 2024;331(12):1007–8. 38421659 10.1001/jama.2024.2252

[3] Lincoff AM, Brown-Frandsen K, Colhoun HM, Deanfield J, Emerson SS, Esbjerg S. Semaglutide and cardiovascular outcomes in obesity without diabetes. N Engl J Med. 2023;389(24):2221–32. 37952131 10.1056/NEJMoa2307563

[4] Drucker DJ. GLP-1-based therapies for diabetes, obesity and beyond. Nat Rev Drug Discov. 2025;24(8):631–50. doi: 10.1038/s41573-025-01183-8 40281304

[5] Jeyakumar Y, Richardson L, Sarma S, Retnakaran R, Kramer CK. Representation of racialised and ethnically diverse populations in multicentre randomised controlled trials of GLP-1 medicines for obesity: a systematic review and meta-analysis of gaps. BMJ Glob Health. 2024;9(11):e017177. doi: 10.1136/bmjgh-2024-017177 39608857 PMC11603712

[6] WHO Expert Consultation. Appropriate body-mass index for Asian populations and its implications for policy and intervention strategies. Lancet. 2004;363(9403):157–63. 14726171 10.1016/S0140-6736(03)15268-3

[7] Caleyachetty R, Barber TM, Mohammed NI, Cappuccio FP, Hardy R, Mathur R, et al. Ethnicity-specific BMI cutoffs for obesity based on type 2 diabetes risk in England: a population-based cohort study. Lancet Diabetes Endocrinol. 2021;9(7):419–26. doi: 10.1016/S2213-8587(21)00088-7 33989535 PMC8208895

[8] Sweatt K, Garvey WT, Martins C. Strengths and Limitations of BMI in the Diagnosis of Obesity: What is the Path Forward?. Curr Obes Rep. 2024;13(3):584–95. doi: 10.1007/s13679-024-00580-1 38958869 PMC11306271

[9] Sun Z, Zheng Y. Metabolic diseases in the East Asian populations. Nat Rev Gastroenterol Hepatol. 2025;22(7):500–16. doi: 10.1038/s41575-025-01058-8 40200111

[10] Kanaya AM. Diabetes in South Asians: uncovering novel risk factors with longitudinal epidemiologic data: Kelly West Award Lecture 2023. Diabetes Care. 2024;47(1):7–16. 38117990 10.2337/dci23-0068 PMC10733655

[11] Yoo SGK, Teufel F, Theilmann M, Si Y, Toure EA, Aryal K, et al. GLP-1 receptor agonists for obesity: eligibility across 99 countries. Lancet Diabetes Endocrinol. 2026;14(2):105–8. doi: 10.1016/S2213-8587(25)00356-0 41519598 PMC12923076

[12] Gupta SK. Implications of ICH-E5: Assessment of drug’s sensitivity to ethnic factors and necessity of a bridging study for global drug development. Perspect Clin Res. 2011;2(4):121–3. doi: 10.4103/2229-3485.86874 22145121 PMC3227328

[13] Lu H, Klopp-Schulze L, Mukker JK, Li D, Kuroki Y, Bolleddula J, et al. Asia-inclusive drug development leveraging principles of ICH E5 and E17 guidelines: Case studies illustrating quantitative clinical pharmacology as a foundational enabler. Clin Transl Sci. 2024;17(10):e70050. doi: 10.1111/cts.70050 39445632 PMC11500040

[14] Lavalle Cobo A, Masson W, Lobo M, Barbagelata L, Forte E, Corral P, et al. Ethnic/Racial and Geographic Disparities on Major Cardiovascular Events in Glucagon Like Peptide-1 receptor Agonists Trials: A Meta-Analysis. Curr Probl Cardiol. 2023;48(11):101940. doi: 10.1016/j.cpcardiol.2023.101940 37422042

[15] Bajaj SS, Zhong A, Zhang AL, Stanford FC. Body Mass Index Thresholds for Asians: A Race Correction in Need of Correction?. Ann Intern Med. 2024;177(8):1127–9. doi: 10.7326/M24-0161 39038288 PMC11707652

[16] von Elm E, Altman DG, Egger M, Pocock SJ, Gøtzsche PC, Vandenbroucke JP, et al. The Strengthening the Reporting of Observational Studies in Epidemiology (STROBE) statement: guidelines for reporting observational studies. Lancet. 2007;370(9596):1453–7. doi: 10.1016/S0140-6736(07)61602-X 18064739

[17] Califf RM, Zarin DA, Kramer JM, Sherman RE, Aberle LH, Tasneem A. Characteristics of clinical trials registered in ClinicalTrials.gov, 2007-2010. JAMA. 2012;307(17):1838–47. 22550198 10.1001/jama.2012.3424 PMC13431740

[18] Gwira JA, Fryar CD, Gu Q. Prevalence of Total, Diagnosed, and Undiagnosed Diabetes in Adults: United States, August 2021-August 2023. 2024. 1–8. 10.15620/cdc/165794 PMC12035661 40085919

[19] Kweon S, Kim Y, Jang M, Kim Y, Kim K, Choi S, et al. Data resource profile: the Korea National Health and Nutrition Examination Survey (KNHANES). Int J Epidemiol. 2014;43(1):69–77. doi: 10.1093/ije/dyt228 24585853 PMC3937975

[20] Huh KY, Song I. Cross-sectional analysis of sociodemographic factors associated with self-reported and knowledge-based health literacy in Korea using data from KNHANES 2023. Sci Rep. 2025;15(1):32297. 40897741 10.1038/s41598-025-08705-9 PMC12405510

[21] Chin GC, Potter AW, Friedl KE. Body mass index is a barrier to obesity treatment. Front Endocrinol (Lausanne). 2024;15:1444568. doi: 10.3389/fendo.2024.1444568 39149118 PMC11324493

[22] Araneta MRG, Kanaya AM, Hsu WC, Chang HK, Grandinetti A, Boyko EJ, et al. Optimum BMI cut points to screen asian americans for type 2 diabetes. Diabetes Care. 2015;38(5):814–20. doi: 10.2337/dc14-2071 25665815 PMC4407753

[23] Hsu WC, Araneta MRG, Kanaya AM, Chiang JL, Fujimoto W. BMI cut points to identify at-risk Asian Americans for type 2 diabetes screening. Diabetes Care. 2015;38(1):150–8. doi: 10.2337/dc14-2391 25538311 PMC4392932

[24] Jeong S-M, Lee DH, Rezende LFM, Giovannucci EL. Different correlation of body mass index with body fatness and obesity-related biomarker according to age, sex and race-ethnicity. Sci Rep. 2023;13(1):3472. doi: 10.1038/s41598-023-30527-w 36859451 PMC9977890

[25] Morey BN, Ryu S, Shi Y, Park HW, Lee S. Acculturation and Cardiometabolic Abnormalities Among Chinese and Korean Americans. J Racial Ethn Health Disparities. 2023;10(4):1605–15. doi: 10.1007/s40615-022-01347-x 35705844 PMC9200372

[26] Li Z, Daniel S, Fujioka K, Umashanker D. Obesity among Asian American people in the United States: A review. Obesity (Silver Spring). 2023;31(2):316–28. doi: 10.1002/oby.23639 36695056 PMC10108164

[27] Haam JH, Kim BT, Kim EM, Kwon H, Kang JH, Park JH, et al. Diagnosis of obesity: 2022 update of clinical practice guidelines for obesity by the Korean Society for the Study of Obesity. J Obes Metab Syndr. 2023. 10.7570/jome23031 37340518

[28] Lim S, Buranapin S, Bao X, Quiroga M, Park KH, Kang J-H, et al. Once-weekly semaglutide 2·4 mg in an Asian population with obesity, defined as BMI ≥25 kg/m2, in South Korea and Thailand (STEP 11): a randomised, double-blind, placebo-controlled, phase 3 trial. Lancet Diabetes Endocrinol. 2025;13(10):838–47. doi: 10.1016/S2213-8587(25)00164-0 40825340

[29] Bray GA. Beyond BMI. Nutrients. 2023;15(10):2254. 37242136 10.3390/nu15102254 PMC10223432

