# Supplementary material for: Fixed BMI eligibility criteria for GLP-1 receptor agonist trials and estimated trial-eligible proportions in Asian and non-Asian populations: A cross-sectional analysis
Source: PLoS One. 2026 Jun 25;21(6):e0351415. doi: 10.1371/journal.pone.0351415 (PMC13298741; doi:10.1371/journal.pone.0351415)

**S1 Table. Hybrid Methodology for Structured Eligibility Criteria Extraction**

The extraction of quantitative and categorical eligibility criteria from the free-text fields of *ClinicalTrials.gov* was performed using a two-stage hybrid approach. This methodology integrates the high-throughput processing capabilities of Large Language Models (LLMs) with the clinical rigor of expert manual curation.

**Stage 1:** Automated Parsing with Large Language Model (Claude / Anthropic)
Approach: Token-level semantic parsing.
Strategy: Few-shot prompting with structured output (JSON).

**Stage 2: Interactive Expert Curation**To ensure 100% accuracy for clinical analysis, all LLM-parsed results were reviewed by clinical experts using a custom-built Interactive Curation Tool.

**Curation workflow**

1. Visual Verification: The tool automatically maps parsed JSON values back to the original source text, highlighting the specific segments (blue markers) used for extraction.

2. Context Validation: Curators verify that numerical cutoffs are correctly associated with their respective operators (e.g., distinguishing between “BMI < 30 excluded” vs “BMI >= 30 required”).

3. Manual Correction: If an automated extraction is missing or incorrect, curators add manual notes and corrections directly within the interface.

4. Export Logic: Only trials verified with explicit manual comments or validated alignments are included in the final feedback loop to ensure the highest data quality.

Below is a representative screenshot of the Expert Curation Dashboard used in this study (NCT02198209 as an example).


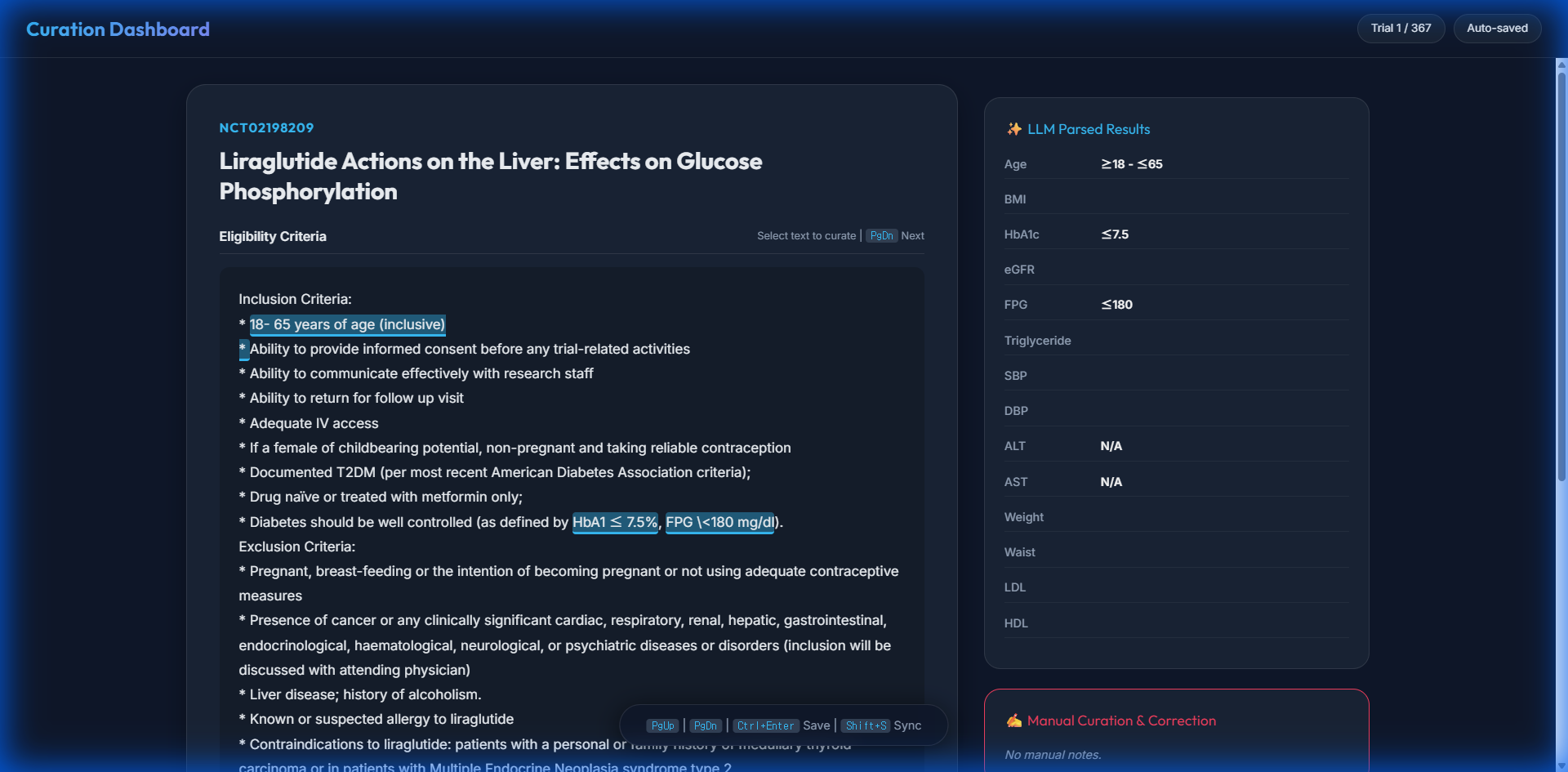

Supplement: S1 Table — (DOCX) [file pone.0351415.s001.docx]
