# Supplementary material for: Fixed BMI eligibility criteria for GLP-1 receptor agonist trials and estimated trial-eligible proportions in Asian and non-Asian populations: A cross-sectional analysis
Source: PLoS One. 2026 Jun 25;21(6):e0351415. doi: 10.1371/journal.pone.0351415 (PMC13298741; doi:10.1371/journal.pone.0351415)
Supplement: S2 Table — (DOCX) [file pone.0351415.s002.docx]

**S2 Table. Measurements and Laboratory Methods**

BMI was calculated as weight in kilograms divided by height in meters squared. BMI was categorized according to the World Health Organization classification: underweight (<18.5 kg/m^2^), normal weight (18.5–24.9 kg/m^2^), overweight (25.0–29.9 kg/m^2^), obesity class I (30.0–34.9 kg/m^2^), obesity class II (35.0–39.9 kg/m^2^), and obesity class III (≥40.0 kg/m^2^). Waist circumference was measured at the midpoint between the lower border of the rib cage and the iliac crest. Elevated waist circumference was defined using population-specific criteria: ≥102 cm for men and ≥88 cm for women in U.S. populations per NCEP ATP III criteria, and ≥90 cm for men and ≥85 cm for women in the Korean population per Korean Society for the Study of Obesity criteria.

Glycated hemoglobin (HbA1c) was measured using standardized high-performance liquid chromatography methods and categorized as normal (<5.7%), prediabetes (5.7–6.4%), or diabetes (≥6.5%) per American Diabetes Association criteria. Fasting plasma glucose was measured in mg/dL and categorized as normal (<100 mg/dL), prediabetes (100–125 mg/dL), or diabetes (≥126 mg/dL) per American Diabetes Association criteria.

Lipid levels were measured using standardized enzymatic methods. Dyslipidemia risk factors were defined based on NCEP ATP III criteria: high total cholesterol (≥240 mg/dL), low HDL cholesterol (<40 mg/dL for men, <50 mg/dL for women), high LDL cholesterol (≥160 mg/dL), and high triglycerides (≥200 mg/dL). Laboratory-defined dyslipidemia was defined as meeting any one of these criteria.

Estimated glomerular filtration rate (eGFR) was calculated using the CKD-EPI 2021 equation without the race coefficient. Chronic kidney disease stages were defined as G1 (≥90 mL/min/1.73m^2^), G2 (60–89 mL/min/1.73m^2^), G3a (45–59 mL/min/1.73m^2^), G3b (30–44 mL/min/1.73m^2^), G4 (15–29 mL/min/1.73m^2^), and G5 (<15 mL/min/1.73m^2^).

Liver transaminases (ALT and AST) were measured using standardized automated analyzers. Elevated liver enzymes were defined as values exceeding the upper limit of normal (>40 U/L).

Blood pressure was measured using standardized protocols, with the average of available readings used for analysis. Hypertension was defined as systolic blood pressure ≥140 mmHg or diastolic blood pressure ≥90 mmHg.

Self-reported hypertension was defined as an affirmative response to physician-diagnosed high blood pressure. Self-reported dyslipidemia was defined as an affirmative response to physician-diagnosed high cholesterol or dyslipidemia.

**NHANES 2021-2023 (United States)**

| **Analyte** | **Method** | **Instrument** | **Reagent/Kit** |
| --- | --- | --- | --- |
| Glycated hemoglobin (HbA1c) | High Performance Liquid Chromatography (HPLC) (Ion-exchange) | Tosoh G8 Analyzer → Bio-Rad D-100 ᵃ (Tosoh/Japan, Bio-Rad/USA) | TSKgel G8 Variant HSi Column (Tosoh/Japan) |
| Fasting plasma glucose | Enzymatic hexokinase method | Roche Cobas c311 (Roche/Germany) | Glucose HK Gen.3 (Roche/Germany) |
| Total cholesterol | Enzymatic method (Cholesterol oxidase) | Roche Cobas 6000 → Cobas 8000 ᵇ (Roche/Germany) | CHOL2 (Roche/Germany) |
| HDL cholesterol | Homogeneous enzymatic colorimetric method | Roche Cobas 6000 → Cobas 8000 ᵇ (Roche/Germany) | HDLC4 (Roche/Germany) |
| LDL cholesterol | Calculated ᶜ (Martin-Hopkins or NIH Eq. 2) | N/A | N/A |
| Triglycerides | Enzymatic method (Glycerol phosphate oxidase) | Roche Cobas 6000 → Cobas 8000 ᵇ (Roche/Germany) | TRIGL (Roche/Germany) |
| Serum creatinine | Kinetic alkaline picrate (Jaffe, IDMS-traceable) | Roche Cobas 6000 → Cobas 8000 ᵇ (Roche/Germany) | CREA2 (Roche/Germany) |
| AST | UV method without P5P (IFCC reference method) | Roche Cobas 6000 → Cobas 8000 ᵇ (Roche/Germany) | ASTL (Roche/Germany) |
| ALT | UV method without P5P (IFCC reference method) | Roche Cobas 6000 → Cobas 8000 ᵇ (Roche/Germany) | ALTL (Roche/Germany) |

**ᵃ** Instrument changed from Tosoh G8 to Bio-Rad D-100 during the survey cycle. Bridging study showed correlation r = 0.999 with mean difference of 2.3%.
**ᵇ** Instrument upgraded from Cobas 6000 to Cobas 8000 during the survey cycle. Bridging studies confirmed correlation r > 0.99 for all analytes.
**ᶜ** LDL cholesterol calculated from total cholesterol, HDL cholesterol, and triglycerides using the Martin-Hopkins equation or NIH Equation 2.
**Source:** NHANES Laboratory Procedure Manuals, CDC/NCHS. Available at: https://wwwn.cdc.gov/nchs/nhanes/continuousnhanes/labmethods.aspx?Cycle=2021-2023

**KNHANES 2021-2023 (Korea)**

| **Analyte** | **Method** | **Instrument** | **Reagent/Kit** |
| --- | --- | --- | --- |
| Glycated hemoglobin (HbA1c) | High Performance Liquid Chromatography (HPLC) | Tosoh G8 (Tosoh/Japan) | HLC-723G8 HbA1c reagent (Tosoh/Japan) |
| Fasting plasma glucose | Enzymatic hexokinase method | Labospect 008AS (Hitachi/Japan) | Qualigent GLU (Sekisui/Japan) |
| Total cholesterol | Enzymatic method | Labospect 008AS (Hitachi/Japan) | Qualigent CHO (Sekisui/Japan) |
| HDL cholesterol | Enzymatic method (Direct) | Labospect 008AS (Hitachi/Japan) | Qualigent HDL (Sekisui/Japan) |
| LDL cholesterol | Enzymatic method (Direct) | Labospect 008AS (Hitachi/Japan) | Qualigent LDL (Sekisui/Japan) |
| Triglycerides | Enzymatic method | Labospect 008AS (Hitachi/Japan) | Qualigent TG (Sekisui/Japan) |
| Serum creatinine | Kinetic colorimetric assay (Jaffe) | Cobas 8000 e602 (Roche/Germany) | CREA (Roche/Germany) |
| AST | UV without P5P (JSCC reference method) | Labospect 008AS (Hitachi/Japan) | Qualigent AST-L (Sekisui/Japan) |
| ALT | UV without P5P (JSCC reference method) | Labospect 008AS (Hitachi/Japan) | Qualigent ALT-L (Sekisui/Japan) |

**Source:** Korea National Health and Nutrition Examination Survey (KNHANES) Raw Data Utilization Guidelines, 2019-2023. Korea Disease Control and Prevention Agency. Available at: https://knhanes.kdca.go.kr/knhanes/dataAnlsGd/utztnGd.do

**Abbreviations:** ALT, alanine aminotransferase; AST, aspartate aminotransferase; HbA1c, glycated hemoglobin; HDL, high-density lipoprotein; HPLC, high-performance liquid chromatography; IDMS, isotope dilution mass spectrometry; IFCC, International Federation of Clinical Chemistry; JSCC, Japan Society of Clinical Chemistry; LDL, low-density lipoprotein; N/A, not applicable; P5P, pyridoxal-5-phosphate; UV, ultraviolet.
