# Supplementary material for: Fixed BMI eligibility criteria for GLP-1 receptor agonist trials and estimated trial-eligible proportions in Asian and non-Asian populations: A cross-sectional analysis
Source: PLoS One. 2026 Jun 25;21(6):e0351415. doi: 10.1371/journal.pone.0351415 (PMC13298741; doi:10.1371/journal.pone.0351415)
Supplement: S3 Table — (DOCX) [file pone.0351415.s003.docx]

**S3 Table. Population Eligibility Rates by Trial Phase and Drug**

| **Drug** | **No. of Trials** | **Non-Asian US, %** | **Asian US, %** | **Korean, %** |
| --- | --- | --- | --- | --- |
| **Early Phase** | 143 | 44.8 (27.7) | 41.2 (29.4) | 37.0 (30.1) |
| Exenatide (2005) | 10 | 60.6 (27.5) | 62.5 (25.3) | 64.4 (26.4) |
| Liraglutide (2010) | 13 | 54.0 (25.1) | 47.5 (29.4) | 40.3 (33.5) |
| Dulaglutide (2014) | 2 | 44.2 (58.4) | 33.6 (43.4) | 30.3 (40.1) |
| Lixisenatide (2016) | 2 | 29.0 (41.0) | 27.7 (39.2) | 31.4 (44.4) |
| Semaglutide (2017) | 26 | 42.7 (19.6) | 35.7 (18.8) | 28.5 (18.2) |
| Tirzepatide (2022) | 16 | 39.5 (28.9) | 35.4 (29.6) | 31.3 (29.1) |
| GLP-1 RA (generic) | 5 | 59.5 (24.1) | 62.5 (22.2) | 58.5 (25.7) |
| Others | 69 | 42.2 (29.3) | 39.4 (32.1) | 35.6 (31.9) |
| **Late Phase** | 141 | 31.9 (32.2) | 26.3 (31.7) | 23.1 (32.1) |
| Exenatide (2005) | 10 | 23.0 (27.3) | 21.3 (28.2) | 18.4 (28.7) |
| Liraglutide (2010) | 31 | 40.1 (31.9) | 31.9 (33.2) | 27.4 (33.7) |
| Dulaglutide (2014) | 7 | 42.7 (46.2) | 43.3 (47.5) | 42.9 (48.8) |
| Lixisenatide (2016) | 5 | 5.8 (1.9) | 6.1 (1.8) | 3.8 (1.8) |
| Semaglutide (2017) | 38 | 33.5 (34.8) | 26.3 (32.7) | 23.5 (33.2) |
| Tirzepatide (2022) | 10 | 12.0 (17.2) | 7.3 (8.1) | 5.1 (5.1) |
| GLP-1 RA (generic) | 13 | 27.6 (28.2) | 24.9 (29.2) | 21.5 (29.8) |
| Others | 27 | 35.2 (31.7) | 29.1 (32.6) | 25.1 (32.8) |
| **Not specified** | 68 | 70.8 (35.8) | 67.8 (39.2) | 66.2 (41.1) |
| Exenatide (2005) | 1 | 66.1 (NA) | 73.8 (NA) | 69.7 (NA) |
| Liraglutide (2010) | 12 | 48.6 (35.5) | 38.1 (39.6) | 35.0 (42.4) |
| Dulaglutide (2014) | 3 | 28.9 (41.2) | 31.2 (45.2) | 29.2 (43.5) |
| Semaglutide (2017) | 16 | 99.9 (0.4) | 100.0 (0.1) | 100.0 (0.1) |
| GLP-1 RA (generic) | 11 | 55.8 (36.6) | 50.2 (38.2) | 46.9 (40.0) |
| Others | 25 | 74.7 (33.9) | 73.4 (36.3) | 72.3 (38.0) |

Data are presented as mean (SD) of population eligibility rates across trials. Eligibility rates were calculated using NHANES 2021-2023 (Non-Asian US, Asian US) and KNHANES 2021-2023 (Korean) data with survey weights.

Early Phase includes Phase 1, Phase 1/2, and Phase 2 trials. Late Phase includes Phase 3, Phase 3/4, and Phase 4 trials. Not specified includes trials without phase information in the registry.

Drug categories are based on the primary GLP-1 receptor agonist mentioned in the trial title. Years in parentheses indicate FDA approval year for type 2 diabetes indication. Tirzepatide is a dual GIP/GLP-1 receptor agonist.

Abbreviations: GIP, glucose-dependent insulinotropic polypeptide; GLP-1 RA, glucagon-like peptide-1 receptor agonist.
