# Supplementary material for: Fixed BMI eligibility criteria for GLP-1 receptor agonist trials and estimated trial-eligible proportions in Asian and non-Asian populations: A cross-sectional analysis
Source: PLoS One. 2026 Jun 25;21(6):e0351415. doi: 10.1371/journal.pone.0351415 (PMC13298741; doi:10.1371/journal.pone.0351415)
Supplement: S4 Table — (DOCX) [file pone.0351415.s004.docx]

**S4 Table. Country distribution of the 352 GLP-1 RA trials.**

| **Country** | **Trials, No.** | **% of 352** | **Region** |
| --- | --- | --- | --- |
| United States | 141 | 40.1 | North America |
| Germany | 53 | 15.1 | Europe |
| China | 49 | 13.9 | East Asia |
| Canada | 38 | 10.8 | North America |
| United Kingdom | 37 | 10.5 | Europe |
| Denmark | 29 | 8.2 | Europe |
| Japan | 28 | 8.0 | East Asia |
| Poland | 24 | 6.8 | Europe |
| Spain | 23 | 6.5 | Europe |
| Mexico | 22 | 6.2 | North America |
| Puerto Rico | 20 | 5.7 | North America |
| Russia | 20 | 5.7 | Europe |
| India | 20 | 5.7 | South Asia |
| Italy | 19 | 5.4 | Europe |
| Netherlands | 17 | 4.8 | Europe |
| Hungary | 17 | 4.8 | Europe |
| Greece | 17 | 4.8 | Europe |
| Taiwan | 17 | 4.8 | East Asia |
| Austria | 16 | 4.5 | Europe |
| Brazil | 16 | 4.5 | South America |
| Sweden | 16 | 4.5 | Europe |
| Israel | 16 | 4.5 | Middle East/North Africa |
| France | 15 | 4.3 | Europe |
| Czechia | 15 | 4.3 | Europe |
| Argentina | 14 | 4.0 | South America |
| South Korea | 14 | 4.0 | East Asia |
| Slovakia | 13 | 3.7 | Europe |
| Belgium | 13 | 3.7 | Europe |
| Romania | 12 | 3.4 | Europe |
| Australia | 11 | 3.1 | Oceania |
| Ukraine | 11 | 3.1 | Europe |
| South Africa | 11 | 3.1 | Africa |
| Bulgaria | 10 | 2.8 | Europe |
| Turkey (Türkiye) | 10 | 2.8 | Middle East/North Africa |
| Malaysia | 8 | 2.3 | Southeast Asia |
| Serbia | 7 | 2.0 | Europe |
| Finland | 7 | 2.0 | Europe |
| Ireland | 6 | 1.7 | Europe |
| Algeria | 5 | 1.4 | Africa |
| Hong Kong | 5 | 1.4 | East Asia |
| New Zealand | 5 | 1.4 | Oceania |
| Switzerland | 5 | 1.4 | Europe |
| Thailand | 5 | 1.4 | Southeast Asia |
| Portugal | 5 | 1.4 | Europe |
| Latvia | 4 | 1.1 | Europe |
| Croatia | 4 | 1.1 | Europe |
| Slovenia | 4 | 1.1 | Europe |
| United Arab Emirates | 3 | 0.9 | Middle East/North Africa |
| Singapore | 3 | 0.9 | Southeast Asia |
| Norway | 3 | 0.9 | Europe |
| Estonia | 3 | 0.9 | Europe |
| Colombia | 2 | 0.6 | South America |
| Lebanon | 2 | 0.6 | Middle East/North Africa |
| Kuwait | 2 | 0.6 | Middle East/North Africa |
| North Macedonia | 2 | 0.6 | Other |
| Pakistan | 2 | 0.6 | South Asia |
| Bosnia and Herzegovina | 1 | 0.3 | Other |
| Egypt | 1 | 0.3 | Middle East/North Africa |
| Iran | 1 | 0.3 | Middle East/North Africa |
| Mauritius | 1 | 0.3 | Other |
| Morocco | 1 | 0.3 | Africa |
| Lithuania | 1 | 0.3 | Europe |

Trials with at least one registered site in the country are counted once; percentages use 352 (the full trial cohort) as the denominator. Country information was available for 344/352 trials (97.7%). Source: AACT countries.txt.

**Aggregated by region**

| **Region** | **Country-trial appearances** |
| --- | --- |
| Europe | 426 |
| North America | 221 |
| East Asia | 113 |
| Middle East/North Africa | 35 |
| South America | 32 |
| South Asia | 22 |
| Africa | 17 |
| Oceania | 16 |
| Southeast Asia | 16 |
| Other | 4 |

Each row counts the sum of trial counts across all countries in the region; trials with sites in multiple countries within a region are counted multiple times.
