# Supplementary material for: Fixed BMI eligibility criteria for GLP-1 receptor agonist trials and estimated trial-eligible proportions in Asian and non-Asian populations: A cross-sectional analysis
Source: PLoS One. 2026 Jun 25;21(6):e0351415. doi: 10.1371/journal.pone.0351415 (PMC13298741; doi:10.1371/journal.pone.0351415)
Supplement: S5 Table — (DOCX) [file pone.0351415.s005.docx]

**S5 Table. Population-relevant exclusion criteria after manual review of the eligibility-criteria text of all 352 GLP-1 RA trials.**

| **Category** | **Exclusion, n (%)** | **Inclusion (target), n (%)** | **Not mentioned, n (%)** | **Unsure, n** |
| --- | --- | --- | --- | --- |
| NAFLD / NASH / MAFLD / MASH | 0 (0.0%) | 21 (6.0%) | 331 (94.0%) | 0 |
| Monogenic diabetes (MODY, HNF1A/HNF4/GCK, secondary) | 33 (9.4%) | 1 (0.3%) | 318 (90.3%) | 0 |
| Severe hepatic impairment (cirrhosis, hepatitis B/C, ALT/AST ≥2.5× ULN) | 177 (50.3%) | 3 (0.9%) | 172 (48.9%) | 0 |
| Bariatric or weight-loss surgery | 111 (31.5%) | 9 (2.6%) | 232 (65.9%) | 0 |
| Race / ethnicity-based exclusion | 6 (1.7%) | 2 (0.6%) | 343 (97.4%) | 1 |

Each candidate regular-expression hit was adjudicated by parallel sub-agent review of the full eligibility text and by clinical expert curation, distinguishing true exclusion criteria from cases where the same term appeared as a target indication (e.g., trials of NAFLD/NASH therapy where biopsy- or imaging-confirmed steatohepatitis is an inclusion criterion). Percentages use 352 as the denominator.
