# Supplementary material for: Fixed BMI eligibility criteria for GLP-1 receptor agonist trials and estimated trial-eligible proportions in Asian and non-Asian populations: A cross-sectional analysis
Source: PLoS One. 2026 Jun 25;21(6):e0351415. doi: 10.1371/journal.pone.0351415 (PMC13298741; doi:10.1371/journal.pone.0351415)
Supplement: S6 Table — (DOCX) [file pone.0351415.s006.docx]

**S6 Table. Sex-stratified estimated trial-eligible proportions in NHANES and KNHANES adults, by population.**

| **Eligibility criterion** | **Population** | **Male, % (95% CI)** | **Female, % (95% CI)** |
| --- | --- | --- | --- |
| BMI ≥25 kg/m² | Non-Asian US | 74.1 (71.3–76.9) | 70.2 (67.1–73.3) |
| BMI ≥25 kg/m² | Asian US | 52.9 (42.6–63.1) | 37.3 (26.1–48.6) |
| BMI ≥25 kg/m² | Korea | 44.4 (43.0–45.8) | 28.7 (27.5–30.0) |
| BMI ≥30 kg/m² | Non-Asian US | 39.6 (36.4–42.9) | 42.2 (38.7–45.6) |
| BMI ≥30 kg/m² | Asian US | 16.6 (8.9–24.3) | 10.5 (3.5–17.5) |
| BMI ≥30 kg/m² | Korea | 8.3 (7.5–9.0) | 5.8 (5.2–6.4) |
| HbA1c ≥6.5% | Non-Asian US | 10.8 (9.3–12.3) | 8.6 (7.0–10.1) |
| HbA1c ≥6.5% | Asian US | 9.8 (5.2–14.5) | 5.4 (3.5–7.3) |
| HbA1c ≥6.5% | Korea | 9.6 (8.8–10.3) | 7.0 (6.4–7.5) |
| HbA1c ≥7.0% | Non-Asian US | 7.4 (6.1–8.7) | 5.8 (4.6–7.1) |
| HbA1c ≥7.0% | Asian US | 7.8 (4.0–11.7) | 3.7 (0.7–6.6) |
| HbA1c ≥7.0% | Korea | 5.6 (5.0–6.1) | 3.8 (3.4–4.2) |
| Composite T2D-trial eligibility | Non-Asian US | 4.5 (3.6–5.5) | 3.8 (2.7–4.8) |
| Composite T2D-trial eligibility | Asian US | 1.8 (-0.1–3.8) | 1.3 (0.3–2.2) |
| Composite T2D-trial eligibility | Korea | 2.6 (2.2–3.1) | 1.6 (1.3–1.8) |
| Composite obesity-trial eligibility | Non-Asian US | 27.2 (24.0–30.3) | 28.6 (26.0–31.2) |
| Composite obesity-trial eligibility | Asian US | 11.4 (4.7–18.1) | 8.2 (2.5–14.0) |
| Composite obesity-trial eligibility | Korea | 6.8 (6.1–7.5) | 4.2 (3.7–4.7) |

Weighted prevalence estimates (95% CI) from NHANES 2021–2023 (Non-Asian US, Asian US) and KNHANES 2021–2023 (Korea). Composite T2D-trial eligibility = age 18–75 (NHANES) / 19–75 (KNHANES) AND BMI ≥25 AND HbA1c 7.0–10.0% AND eGFR ≥30 (missing eGFR treated as eligible). Composite obesity-trial eligibility = age 18–65 (NHANES) / 19–65 (KNHANES) AND BMI ≥30 AND HbA1c <6.5 (missing treated as eligible) AND eGFR ≥60 (missing treated as eligible). Composite criteria match the main waterfall analysis in Fig 3.
