# Supplementary material for: Fixed BMI eligibility criteria for GLP-1 receptor agonist trials and estimated trial-eligible proportions in Asian and non-Asian populations: A cross-sectional analysis
Source: PLoS One. 2026 Jun 25;21(6):e0351415. doi: 10.1371/journal.pone.0351415 (PMC13298741; doi:10.1371/journal.pone.0351415)
Supplement: S7 Table — (DOCX) [file pone.0351415.s007.docx]

**S7 Table. Distribution of minimum BMI eligibility thresholds across the 352 GLP-1 RA trials.**

| **Minimum BMI, kg/m²** | **Trials at this exact cutoff** | **% of 352 (exact)** | **Trials with cutoff ≥ this value** | **% of 352 (≥)** |
| --- | --- | --- | --- | --- |
| 23.0 | 20.0 | 5.7 | 136.0 | 38.6 |
| 25.0 | 31.0 | 8.8 | 108.0 | 30.7 |
| 27.0 | 42.0 | 11.9 | 76.0 | 21.6 |
| 27.5 | 0.0 | 0.0 | 34.0 | 9.7 |
| 28.0 | 7.0 | 2.0 | 34.0 | 9.7 |
| 30.0 | 22.0 | 6.2 | 27.0 | 7.7 |
| 32.0 | 1.0 | 0.3 | 5.0 | 1.4 |
| 35.0 | 4.0 | 1.1 | 4.0 | 1.1 |

Only trials with a numeric minimum BMI in their eligibility criteria are counted; trials with BMI specified only as a maximum, range, or qualitative descriptor are excluded. 219 of 352 trials (62.2%) specified a numeric BMI minimum. Note: counts at the exact cutoffs 23 (20 trials) and 25 (31 trials) differ by 1 trial each from the main-text Table 2 values (21 and 32, respectively), reflecting a small curation difference at the 23.0 / 25.0 boundary; the response letter and supporting analyzes use these counts consistently.
