# Supplementary material for: Fixed BMI eligibility criteria for GLP-1 receptor agonist trials and estimated trial-eligible proportions in Asian and non-Asian populations: A cross-sectional analysis
Source: PLoS One. 2026 Jun 25;21(6):e0351415. doi: 10.1371/journal.pone.0351415 (PMC13298741; doi:10.1371/journal.pone.0351415)
Supplement: S8 Table — (DOCX) [file pone.0351415.s008.docx]

**S8 Table. Primary-endpoint classification of the 352 GLP-1 RA trials.**

| **Principal endpoint category** | **Trials, n** | **% of 352** |
| --- | --- | --- |
| HbA1c | 76 | 21.6 |
| Other glycemic (FPG, insulin/C-peptide, time-in-range, diabetes remission) | 37 | 10.5 |
| Body weight, percent change | 26 | 7.4 |
| Body weight, kilograms | 6 | 1.7 |
| Body weight, other measure | 5 | 1.4 |
| BMI change | 1 | 0.3 |
| Waist circumference | 0 | 0.0 |
| Liver / NAFLD / NASH | 11 | 3.1 |
| Cardiovascular (MACE, MI, stroke, heart failure) | 28 | 8.0 |
| Renal (eGFR, albuminuria) | 6 | 1.7 |
| Pharmacokinetic / pharmacodynamic | 42 | 11.9 |
| Safety / adverse events | 47 | 13.4 |
| Quality-of-life / patient-reported | 6 | 1.7 |
| Other / unclassified | 61 | 17.3 |
| No primary outcome rows in AACT | 0 | 0.0 |

Each trial was assigned a single principal-endpoint category based on AACT design_outcomes rows with outcome_type = ‘primary’, reviewed in parallel by six sub-agents using a priority-ordered rubric (cardiovascular > renal > liver > weight % > weight kg > BMI > HbA1c > other glycemic > waist > PK/PD > QoL > weight-other > safety/AE > other). Trials with no primary outcome row in AACT are listed as ‘no primary outcome rows’.
