# Supplementary figures and images for: Fixed BMI eligibility criteria for GLP-1 receptor agonist trials and estimated trial-eligible proportions in Asian and non-Asian populations: A cross-sectional analysis
Source: PLoS One. 2026 Jun 25;21(6):e0351415. doi: 10.1371/journal.pone.0351415 (PMC13298741; doi:10.1371/journal.pone.0351415)

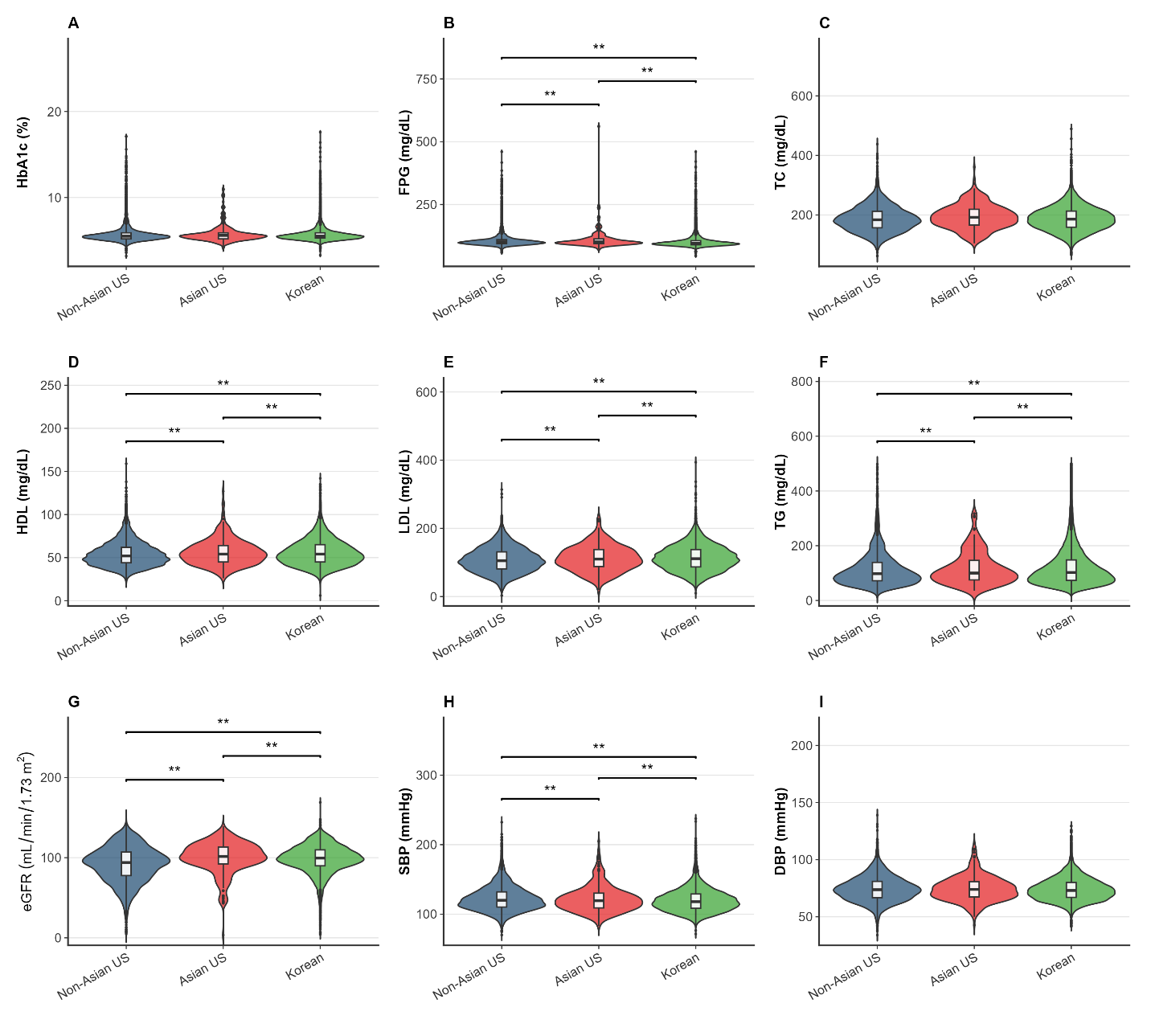

Supplement: S1 Fig — Violin plots showing the distribution of key laboratory parameters across three populations: Non-Asian US (blue), Asian US (red), and Korean (green) adults. Parameters include glycated hemoglobin (HbA1c), fasting plasma glucose, body mass index (BMI), estimated glomerular filtration rate (eGFR), triglycerides, LDL cholesterol, and alanine aminotransferase (ALT). Horizontal dashed lines indicate common eligibility thresholds used in GLP-1 receptor agonist clinical trials. White dots within violins represent median values; thick bars represent interquartile ranges. Survey weights were applied to generate population-representative distributions. Notable differences include substantially higher BMI distributions in Non-Asian US compared with Asian populations, and higher triglyceride levels in Korean adults. (PNG) [file pone.0351415.s009.png]

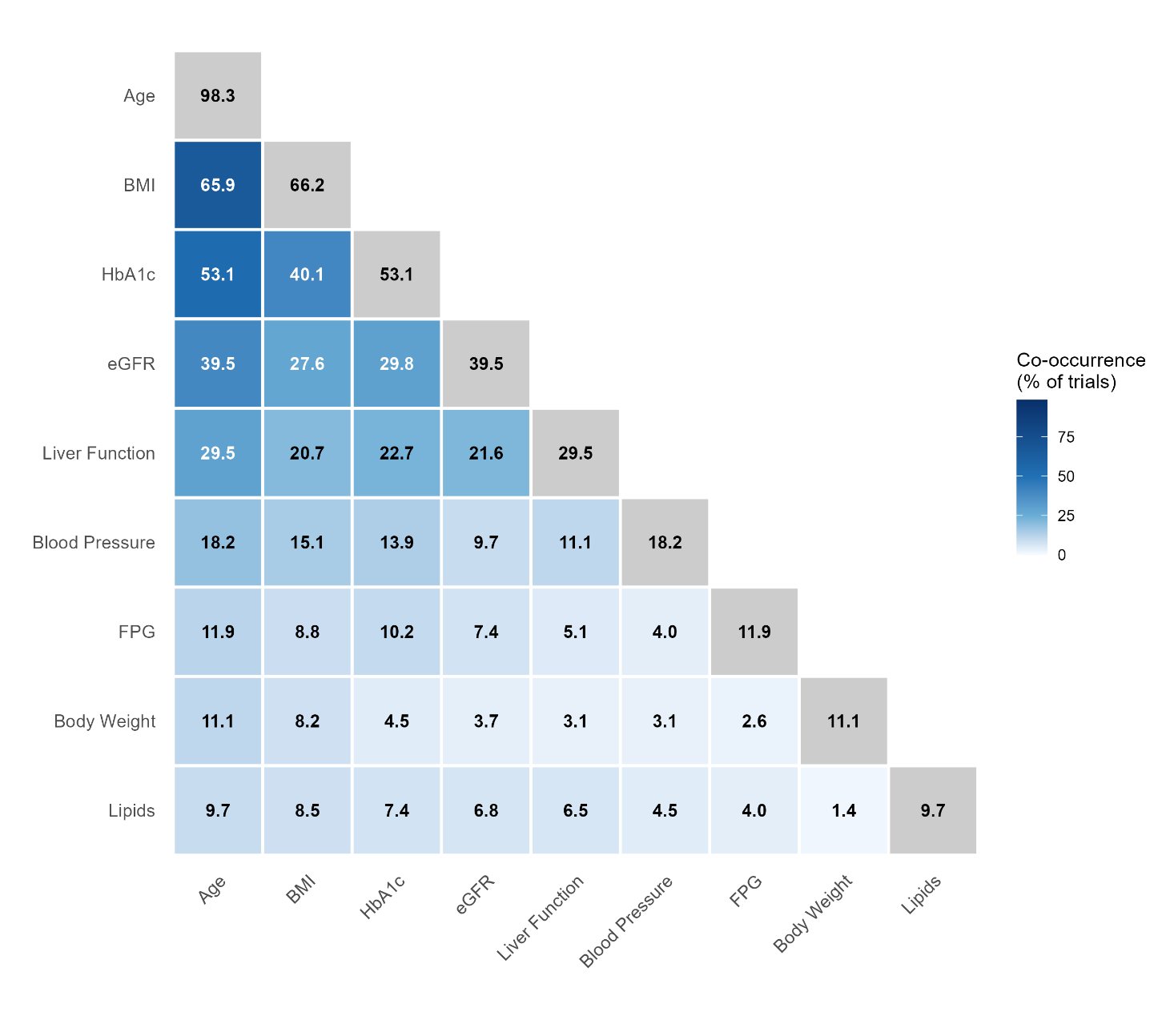

Supplement: S2 Fig — Heatmap showing the percentage of 352 trials in which pairs of eligibility criteria were specified together. Diagonal values (gray) represent the percentage of trials specifying each individual criterion: age (98.3%), BMI (66.2%), HbA1c (53.1%), eGFR (39.5%), liver function (29.5%), blood pressure (18.2%), fasting plasma glucose (11.9%), body weight (11.1%), and lipids (9.7%). Off-diagonal values represent co-occurrence rates. Criteria are ordered by frequency (descending). P values were calculated using chi-squared tests. Abbreviations: ALT, alanine aminotransferase; BMI, body mass index; eGFR, estimated glomerular filtration rate; FPG, fasting plasma glucose; GLP-1 RA, glucagon-like peptide-1 receptor agonist; HbA1c, glycated hemoglobin. (PNG) [file pone.0351415.s010.png]

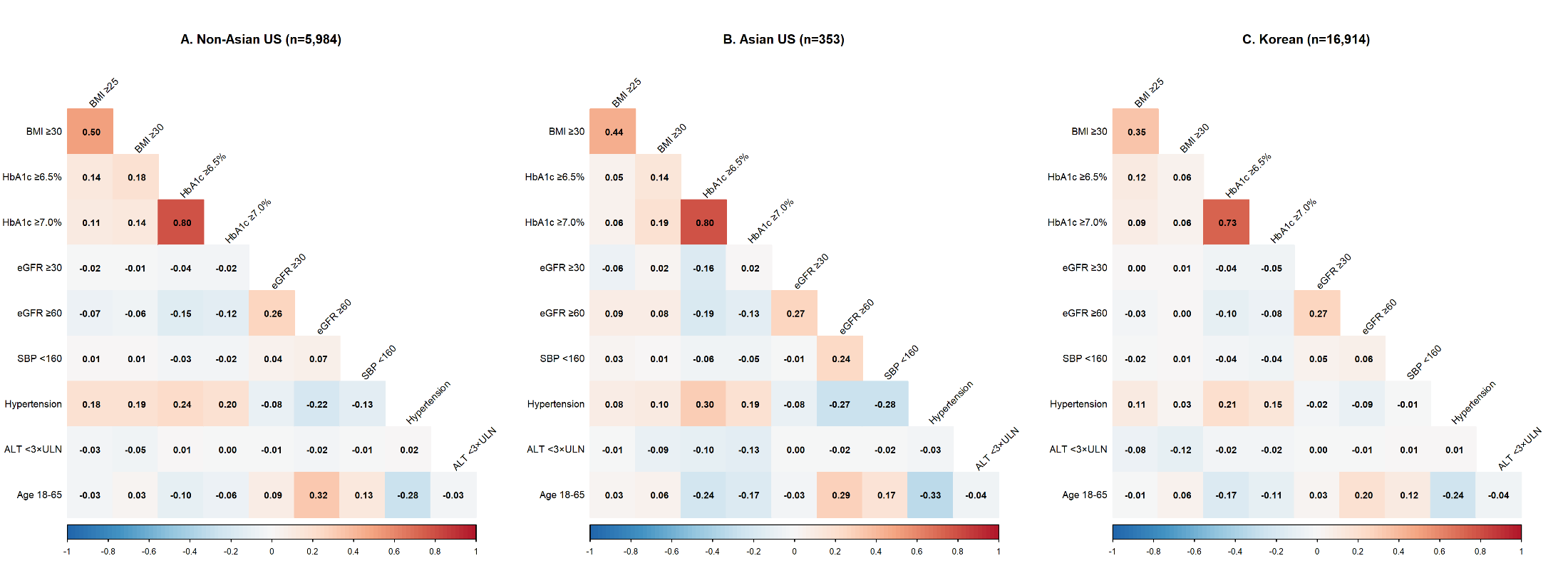

Supplement: S3 Fig — Three-panel heatmap showing tetrachoric correlations between binary eligibility criteria across Non-Asian US, Asian US, and Korean populations. Data are from NHANES 2021−2023 (Non-Asian US and Asian US) and KNHANES 2021−2023 (Korean). Strong positive correlations were observed between HbA1c ≥ 6.5% and HbA1c ≥ 7.0% (r = 0.73–0.80) and between BMI ≥ 25 and BMI ≥ 30 (r = 0.35–0.50), indicating that related criteria track together across populations. Moderate negative correlations were observed between age criteria (18−65 years) and hypertension (r = −0.24 to −0.33), reflecting the age-dependent nature of hypertension prevalence. eGFR criteria (≥30 and ≥60 mL/min/1.73m2) showed consistent positive correlations (r = 0.26–0.27) across all three populations. (PNG) [file pone.0351415.s011.png]
